# A Caregiver Digital Intervention to Support Shared Decision Making in Child and Adolescent Mental Health Services: Development Process and Stakeholder Involvement Analysis

**DOI:** 10.2196/24896

**Published:** 2021-06-15

**Authors:** Shaun Liverpool, Julian Edbrooke-Childs

**Affiliations:** 1 Anna Freud National Centre for Children & Families London United Kingdom; 2 Faculty of Health, Social Care & Medicine Edge Hill University Ormskirk United Kingdom; 3 University College London London United Kingdom

**Keywords:** digital health intervention, caregivers, parents, child mental health

## Abstract

**Background:**

Parents and caregivers are generally recognized by literature and the law as key to child and adolescent mental health decisions. Digital interventions are increasingly being used to support care and treatment in child and adolescent mental health services (CAMHS). However, evidence of the design and development process is generally not made available.

**Objective:**

In light of calls for more transparency, this paper aims to describe the development of an evidence-based, theoretically informed digital decision support intervention for parents and caregivers of young people accessing CAMHS.

**Methods:**

The intervention was developed in line with the UK Medical Research Council framework for developing complex interventions. The process incorporated the steps for developing patient decision aids, as follows: assessing need, assessing feasibility; defining objectives; identifying the framework of decision support; and selecting the methods, designs, and dissemination approach. We synthesized theory, research, international guidelines, and input from relevant stakeholders using an iterative design approach.

**Results:**

The development steps resulted in Power Up for Parents, a decision support intervention, with five key features (ie, decisions, goals, journey, support, and resources). The intervention aims to encourage discussion, allow parents to ask questions during sessions or seek further information between sessions, and allow service providers to tailor the shared decision-making process to accommodate the needs of the parent and child.

**Conclusions:**

We confirmed that it is possible to use input from end users—integrated with theory and evidence—to create digital interventions to be used in CAMHS. Key lessons with implications for practice, policy, and implementation science, along with preliminary findings, are presented.

**International Registered Report Identifier (IRRID):**

RR2-10.2196/14571

## Introduction

### Background

Digital health interventions have been increasingly used in child and adolescent mental health services (CAMHS) [1-3]. Power Up, a mobile phone app for supporting young people in shared decision making (SDM), has shown some evidence of promise; young people who used Power Up reported greater levels of SDM after the intervention period [4,5]. SDM is central to person-centered care and describes a process in which service users and service providers collaborate to make treatment decisions [6]. Depending on the age of the child, parents (including nonbiological primary caregivers) sometimes report feeling excluded from the decision-making process and therefore may also benefit from receiving additional support [7]. Previous research has highlighted that parents’ decision support needs include obtaining information, talking to others, and feeling a sense of control over the decision-making process [8,9].

In addition, parents of children with mental health difficulties report experiencing an *emotional roller coaster* [10]. Furthermore, researchers identified parents’ emotions as a possible influencing factor in the SDM process [11-13]. A review of parent-targeted SDM interventions for use in CAMHS revealed that existing interventions rarely addressed this concern, and only one available intervention explicitly addressed emotional support [14]. Counseling in Dialogue is a face-to-face intervention found to lower decisional conflict and promote the acceptance of recommended treatments [15]. However, concerns about stigma and confidentiality, shame or embarrassment in attending services, financial costs, time, appropriateness, or limited access to services are usually among the many barriers to accessing in-person CAMHS [16,17]. As a result, existing efficacious face-to-face interventions are adopting digital technology as a means of addressing these barriers [18,19]. Three interventions identified in a previous review [14] were considered to be digitally accessible. Two of those interventions targeted parents of children with autism spectrum disorders [20,21], and one intervention targeted parents of children with attention-deficit/hyperactivity disorders [22]. However, recommendations to develop interactive digital interventions that promote well-being factors, in addition to targeted behavior change, are gaining momentum [23].

Despite the growing interest in digital health interventions, detailed descriptions of the development process of digital interventions used in CAMHS are limited [24], with implications for clinical and research reproducibility. Nonetheless, recent reviews of the extant literature have described innovative technological applications in parent management training programs [25,26] and programs to promote child health [27]. Although this research shows great efficacy for the use of parent-targeted technology in child health care, to the best of our knowledge, there are presently no parent-targeted interactive mobile apps designed for and tested in CAMHS that support an affective-appraisal SDM process [14]. The affective-appraisal approach refers to the ability to include key decision makers (ie, child or young person, parents, and service providers) and incorporate and address the influence of parental affective states on the SDM process [13].

Furthermore, health care quality standards and guidelines identify and define SDM as an essential characteristic of good quality care, endorsing support and interventions for both service users and service providers [28]. Experts highlight that for SDM to occur, the process should include the following nine essential elements: patient values and preferences, options, professional knowledge and recommendations, make or explicitly defer a decision, define and explain the problem, check and clarify understanding, explore benefits and risks, discuss patient’s ability and self-efficacy, and arrange follow-up [29]. However, available interventions meet an average of 4.57 (SD 1.93) SDM elements [14]. Furthermore, most of the work on defining, facilitating, and supporting SDM has focused on adult health care and dyad relationships between primary service users and health care providers [29,30]. In CAMHS, parents are sometimes surrogate decision makers or the parent, child, and health care provider engage in a triad decision-making process [13]. Owing to the many perceived challenges associated with decision making in pediatric care, researchers commonly highlight the lack of an evidence-based holistic conceptualization of SDM [31]. Therefore, in line with the broader health literature, it is recommended that all efforts are made to improve SDM. In so doing, experts call for clinicians to recognize SDM as an ethical imperative, stimulate a bidirectional flow of accurate and tailored information, and give patients and their families resources that facilitate an effective SDM process [32].

### Objectives

Given the importance of SDM and the feasibility of digital interventions in CAMHS, it is essential to develop theoretically informed interventions. The overall aim of this paper is to describe the development of an evidence-based digital intervention for use by parents accessing CAMHS. Consequently, the following subobjectives are addressed:

1. Develop a logic model outlining how the intervention is proposed to work.
2. Consolidate evidence-based content to support the affective-appraisal model of SDM.
3. Involve end users in the design and development of an SDM intervention for use in CAMHS.
4. Highlight key learning and recommendations.

## Methods

### Framework for Intervention Development

The UK Medical Research Council (MRC) framework for the development and evaluation of complex interventions was adopted. The intervention was described as complex, in line with the conventional definition describing complex interventions as interventions with several interacting components. The MRC framework proposes that during the development stage, it is important to identify the evidence base, identify the theory, and model the process and outcomes [33]. Alongside the MRC framework, activities are guided by the steps for developing decision aids [34].

### Assessing Need

A broad overview of the literature explored existing evidence for the prevalence of child mental health problems, factors influencing SDM, and potential impact on the family. Another systematic review aimed to better understand the emotional experiences of having a child with mental health problems and explored how those experiences may influence parental involvement in care and treatment decisions (findings submitted for publication). In addition, existing decision support interventions available for parents of children with mental health problems were identified and assessed against SDM elements [14]. Qualitative interviews were also conducted to obtain insight into how clinicians and parents perceived and described experiences of SDM and to identify the support systems used [13].

### Assessing Development Feasibility

First, as this research was part of a PhD project, it was agreed that the 3-year timeline was appropriate to develop and evaluate an intervention. Second, a preexisting relationship with the technology company (Create Health) made it suitable for the development of a digital intervention [5]. In addition, the financial resources necessary to develop the intervention were available through the PhD project funding. Furthermore, preliminary evidence from the original Power Up for young people suggested that it was feasible to develop and evaluate a novel digital intervention for CAMHS [4].

### Defining the Objectives of the Decision Support Tool

On the basis of an overview of the literature and feedback from parents, practitioners, and researchers (described later in the paper), the following primary objectives were considered necessary to guide the intervention’s development process:

1. Encourage discussion (ie, three-talk model proposed by Elwyn et al [35]).
2. Allow parents to ask questions during sessions or seek further information within sessions.
3. Provide a space for parents to identify their feelings and moods and receive support.
4. Allow service providers to tailor the SDM process to accommodate the needs of the parent and child (eg, informed vs involved).

### Identifying the Framework of Decision Support

In general, the development process of the intervention was conducted in line with the International Patient Decision Aids Standards. These guidelines encourage the use of a systematic development process, disclosing conflicts of interest, internet delivery, using plain language, and basing information on up-to-date evidence, among others [36,37]. More specifically, in line with an affective-appraisal approach [13], the Youth SDM model [38], the Integrative Model of SDM in medical encounters, highlighting the nine essential elements of SDM [19] and the Ottawa Decision Support Framework [39] informed the content of the intervention. The Ottawa Decision Support Framework has been used to develop and evaluate over 50 patient decision aids, measures (eg, Decisional Conflict Scale), and training in providing decision support.

The Youth SDM model highlights three key SDM functional areas: setting the stage for youth SDM, facilitating youth SDM, and supporting youth SDM. The authors recommended that setting the stage for youth SDM should involve providing an introduction to the concept of SDM and inviting and acknowledging the service user’s preference for involvement. To facilitate this, a co-design process to develop a webpage to define and explain SDM was undertaken (discussed in the *Stakeholder Involvement* section). Consequently, the webpage became the welcome screen for the intervention to *set the stage* for SDM.

The Integrative Model of SDM was used to *facilitate the SDM process*. The current intervention was designed to incorporate all the nine elements of SDM. Examples are presented in the Results section. In addition, the Ottawa Decision Support Framework was used to inform *support* for the SDM process. The framework proclaims that participants’ decisional needs will affect decision quality, which in turn affects actions or behaviors (eg, delay), health outcomes, emotions (eg, regret or blame), and appropriate use of health services. This framework was pertinent to the intervention, as previous research highlighted the potential impact of parents’ emotions on the SDM process.

### Selecting the Methods, Designs, and Planning for the Feasibility and Pilot Study

#### Overview

The remaining three steps outlined by O’Connor and Jacobsen [34] were collapsed under the subheading *stakeholder involvement*. There is an overarching consensus that involving end users in the development of health interventions is critical for successful implementation. Developers and researchers converge on the understanding that patient and public involvement (PPI) can benefit the uptake and usage of interventions. More specifically, the involvement of end users is known to improve idea generation and creativity [40-42]. The following sections describe how various stakeholders are involved in the development of the intervention.

#### Stakeholder Involvement

##### Steering Committee

From conception, a steering committee was formed comprising a senior researcher, a colleague with experience in the development of digital interventions, and 3 parents with experience of having a child with a mental health problem; the committee was chaired by the primary author (SL). The parents were appointed as part of the steering committee after expressing interest in this study at various presentations undertaken by the primary author. The committee was ideal for consensus forming and was mainly responsible for ensuring that the development process was transparent and unbiased. The steering committee also guided the feasibility and pilot study of the intervention by offering strategies to promote recruitment. Meetings convened on a web-based platform for a total of 6 times throughout the intervention design and development phase.

##### Patient and Public Involvement

The overall objective of the consultations was to obtain parents’ expert advice on the research and intervention design. However, gaining insight into how parents may use digital health interventions and obtaining input on how to improve the intervention before this study began was necessary. First, an email consultation was conducted with the Family Research Advisory Group at the National Children’s Bureau. Information about the aims of this study and plans for an intervention with specific questions to generate ideas were shared with the research team at the National Children’s Bureau. The team contacted 9 parents who provided input on the value of the intervention, what support might be needed, and which group of parents we should target for recruitment. Prototype development was initiated based on the input received. Second, the study design and an example of how the intervention might be used were presented to the group at a scheduled meeting. The pros and cons of digital versus other formats of decision-making tools were discussed along with general thoughts and concerns regarding the study and intervention design. The prototype was refined and updated before the final meeting. At the final meeting**,** a group discussion, including a presentation of the prototype, was conducted to examine the penultimate version of the intervention and study design. There were further discussions on how parents could use and benefit from the intervention in practice. Further refinement of the prototype was carried out based on the feedback received.

##### Showcase Pollinator Event With Clinicians and Researchers

At a showcase pollinator event, which was held in Austria at the Technology Enabled Mental Health Summer School, the prototype was then presented to clinicians, researchers, and intervention developers who were asked to provide feedback and specifically provide input to improve the interactivity of the intervention. Three roundtable discussions followed, and input was obtained from a total of 12 experts in the area of child mental health. Attendees at the event had a specific interest in digital interventions to prevent, treat, and promote policies for children and youth mental health.

##### Public Engagement

A collaborative approach was adopted to develop and design a webpage to promote SDM in CAMHS. First, a survey to elicit the public’s opinion on the preferred mode of delivery for an SDM resource was conducted via social media. Responses from clinicians, parents, children and young people, school staff, and others were in favor of a web resource. Consequently, 3 parent champions and 4 young champions from the Anna Freud National Centre for Children and Families attended two workshops and provided email feedback on two versions of the webpage before agreeing to the final versions. At the first workshop, participants explored what SDM meant, and a consensus was reached for a family friendly definition that could be displayed on the webpage. In the second workshop, participants were involved in designing the paper prototypes of the webpage. Consequently, the webpage was designed, and the content was updated based on the feedback received. The communications team at the Centre was then involved to ensure that the content and design were in line with the Centre’s standards. Consequently, the webpage was presented as the welcome screen for the intervention.

##### App Developers

The app developers at Create Health were responsible for the technical development of the intervention. However, design-specific components such as swipe versus touch features, labels for the settings menu of the app, and data security were proposed by the developers and included only after they were agreed upon by the primary author and the steering committee. On the basis of feedback from the steering committee, PPI sessions, and parent experts, a series of paper prototyping and digital designs were developed before the final version was adopted.

### Ethics

Ethical approval for the development and pilot testing of Power Up for Parents was granted by the University College London and by the London Surrey Research Ethics Committee (IRAS 236277).

## Results

### Evidence Base

The development process highlighted the need for an SDM intervention targeting parents of children with mental health concerns. Decisions could include, but not be limited to, medications, types of therapy, or service needs. The literature reviews revealed a high prevalence of child mental health problems, several decision-making opportunities, barriers to and facilitators of SDM, and positive outcomes when SDM was adopted in care. The potential influence of a parent’s emotional state on the decision-making process was also identified [8-13,43-45]. Quantitative findings also highlighted a large number of parents reporting involvement in SDM and possible associations among ethnicity, their relationship to the child, and the presence of conduct problems or learning difficulties. Nonetheless, parents and service providers expressed the importance of including parents in the decision-making process. The existing parent-targeted decision support tools identified met an average of 4.57 (SD 1.93) SDM elements out of a possible nine elements [14]. Furthermore, that review reported time, accessibility, and appropriateness of the intervention as factors influencing usage and implementation of interventions, providing additional support for a digital mode of delivery. Table 1 presents an overview of how the evidence informed the intervention’s design objectives and key features.

**Table 1:** Overview of the intervention’s objectives and key features.

Research evidence	Intervention design objective	Key features of the intervention
Recognizing the need for help can be challenging, as carers’ perceptions of their child’s mental health difficulties differ from those of their child, teachers and health professionals. These disagreements are reflected in carers reporting not feeling listened to or respected, further adding to frustrations and disappointment.	Encourage discussion Allow parents to ask questions during sessions or seek further information within sessions	Decisions and goals Decisions and resources
Findings suggest that parents are “expected to, but not always able to” engage with CAMHS^a^ due to the “emotional roller coaster” they experience.	Provide a space for parents to identify their feelings or moods and receive support	Support and journey
Findings suggest that the triad relationship is unique and can be challenging in CAMHS. Recommendations are made to explore opportunities for varying levels of involvement, such as “informed” versus “actively involved” parents.	Allow service providers to tailor the SDMb process to accommodate the needs of the parent and child (ie, informed vs involved)	Decisions and resources
Findings indicated that time, accessibility, and the appropriateness of the intervention emerged as factors influencing the usage and implementation of parent-targeted SDM interventions.	Be suitable and accessible to parents	Digital mode of delivery

^a^CAMHS: child and adolescent mental health services.

^b^SDM: shared decision making.

### Logic Model

The abovementioned evidence was explored in detail and presented in a logic model to outline the purpose of the intervention. Multimedia Appendix 1 provides an overview of the adapted Evidence-Based Practice Unit Logic Model [46], consisting of four parts that describe the intervention and target audience. The logic model also highlights the aims of the intervention and expected outcomes once implemented. In addition, a list of potential moderators that may influence usage and implementation were reported.

### Outline of the Intervention

#### Overview

**Figure 1:**
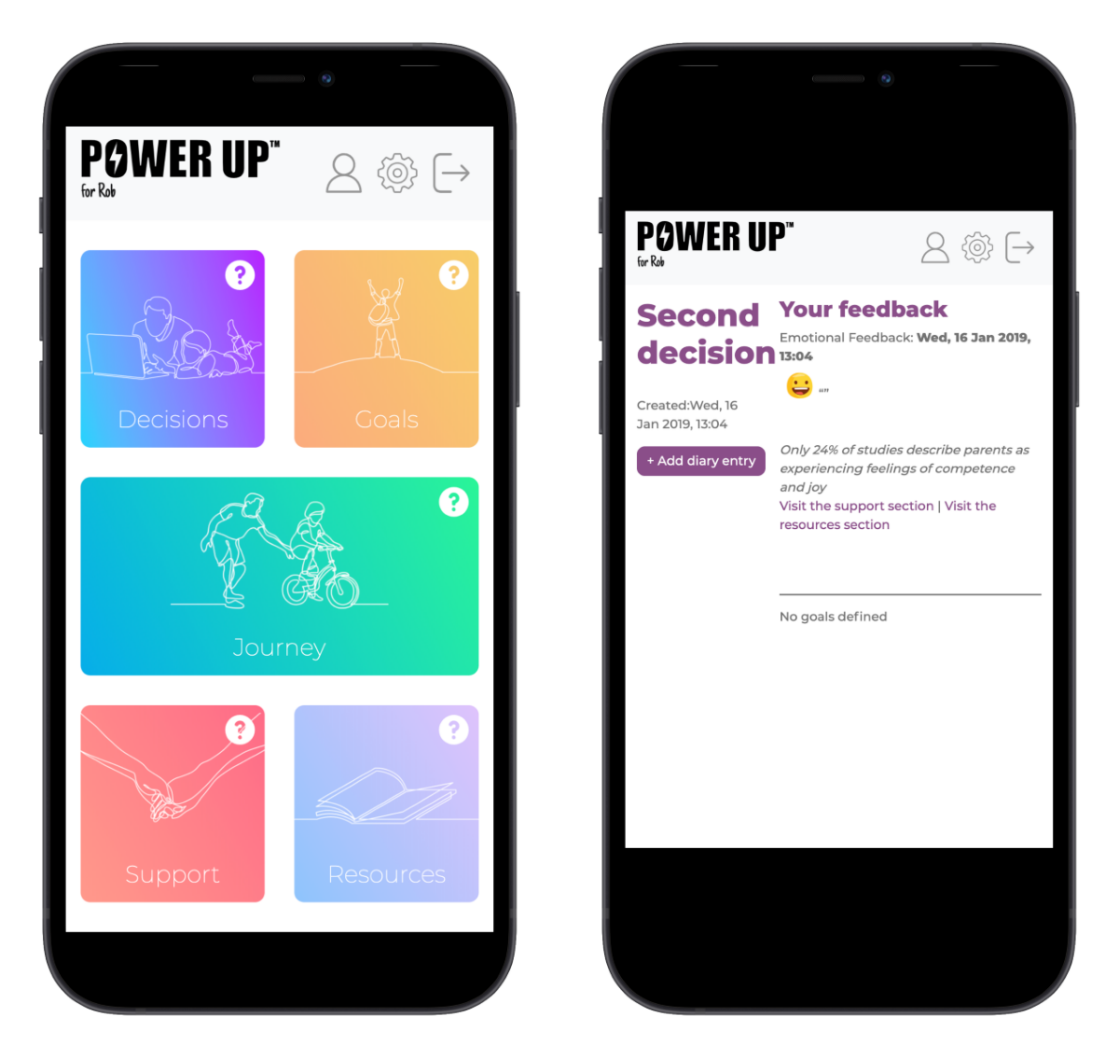
Examples of the home screen and decision tab.

This section summarizes the key features of the resulting prototype and the user manual (Multimedia Appendix 2). The Power Up for Parents title was adopted as this project was an emended version of the original Power Up intervention for young people that supports and promotes SDM in CAMHS [4,5]. Although the current prototype is referred to as Power Up for Parents, feedback from PPI sessions indicated that nonbiological caregivers may feel excluded. In response to this, the prototype included a customization feature to change the word *Parents*. Therefore, it can be labeled *Power Up for Rob* to reflect the child’s or parent’s name (Figure 1). The overall structure of the app content is as follows.

#### Decisions

This is a decision aid that guides users to seek information about treatment options, to review the benefits and risks of each option, to track decisions, and to record where more information or support is needed (Figure 1). In addition, as the research focused on the triad relationship, parents were encouraged to involve others in the decision-making process by seeking preferences from the clinicians, their child, or other relevant persons. This section uses the nine essential elements of SDM to “walk” users through the decision-making process, prompting users to answer questions such as “Do you have sufficient information about the options available to you?” and “ Do you feel ready to make this decision?” The other sections below provide additional support throughout the decision-making process that is in line with the affective-appraisal model of SDM.

#### Goals

**Figure 2:**
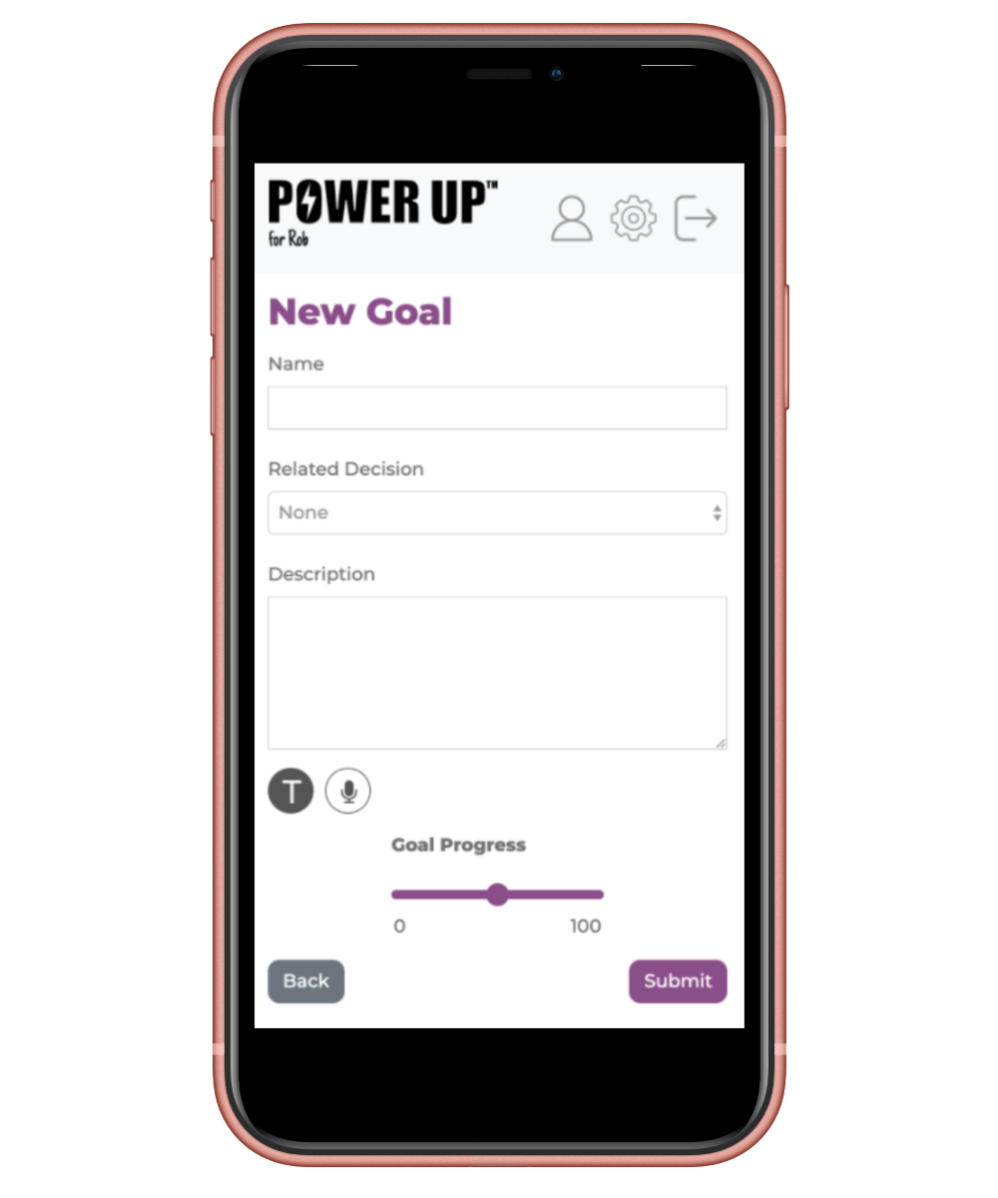
Examples of the goal tab.

This feature is used in sessions or between sessions to record and track goals, as they are discussed with service providers and young service users. It allows users to set individual or consensus goals and explore plans to achieve these goals (Figure 2). In addition, parents could record any questions or concerns to address in the following session. Research findings suggest that goal-setting and tracking progress are associated with higher self-efficacy [47], and this is one approach to promote SDM in CAMHS [48].

#### Journey

**Figure 3:**
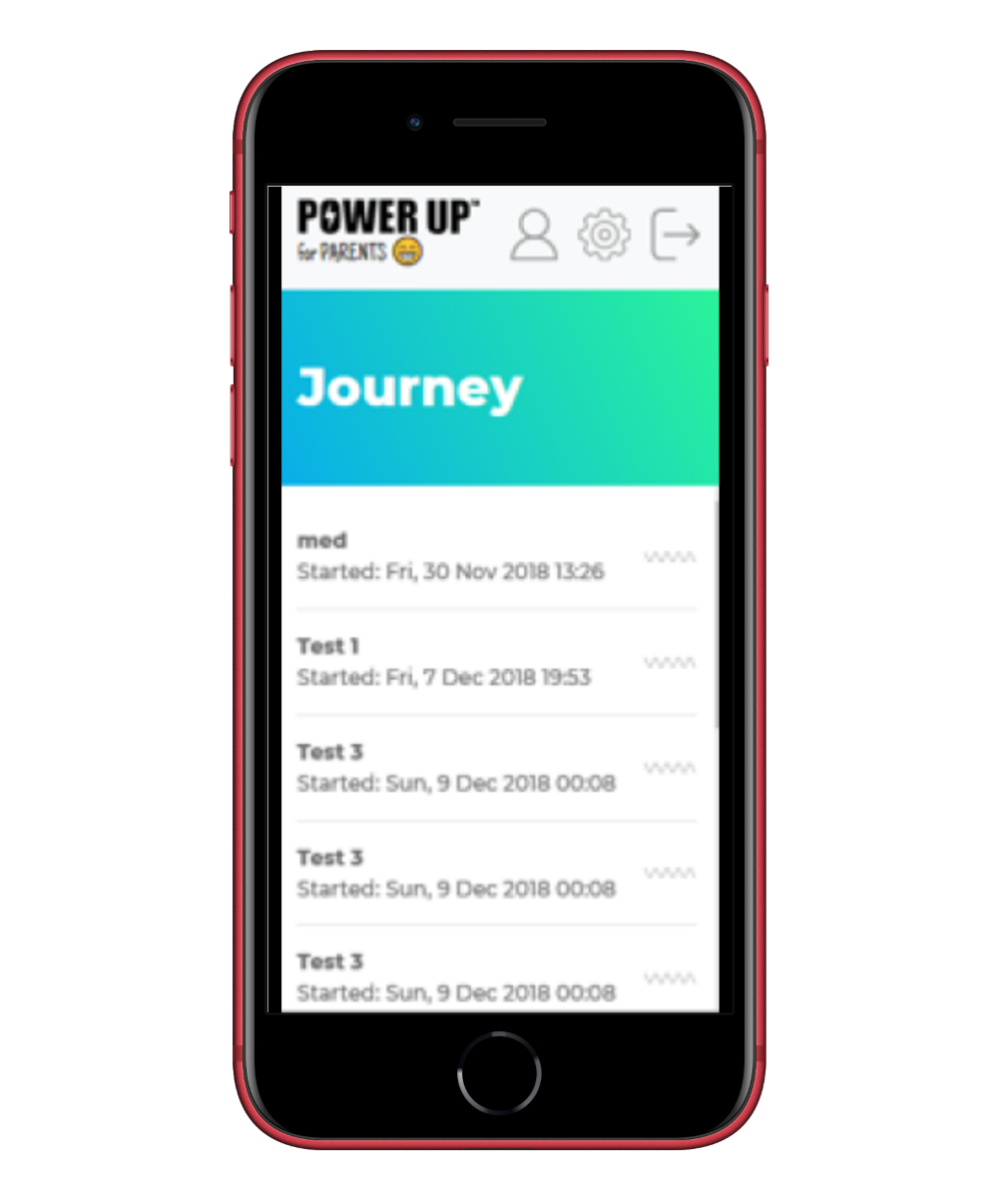
Examples of the journey tab.

This feature allows parents to reflect on their emotions or issues that may affect their decision-making process. A parent could decide to share the content with the child and the clinician, and it could be used during and within sessions to keep track of the decision-making journey from user readiness to outcomes. Expectations, experiences, and reflections are recorded using the diary function (Figure 3). The usefulness of implementing case tracking and documenting client journeys has been highlighted in previous research [49]. Although previously explored in primary care services, those authors highlighted the importance of monitoring the comprehensiveness of service responses and the experiences of clients.

#### Support

**Figure 4:**
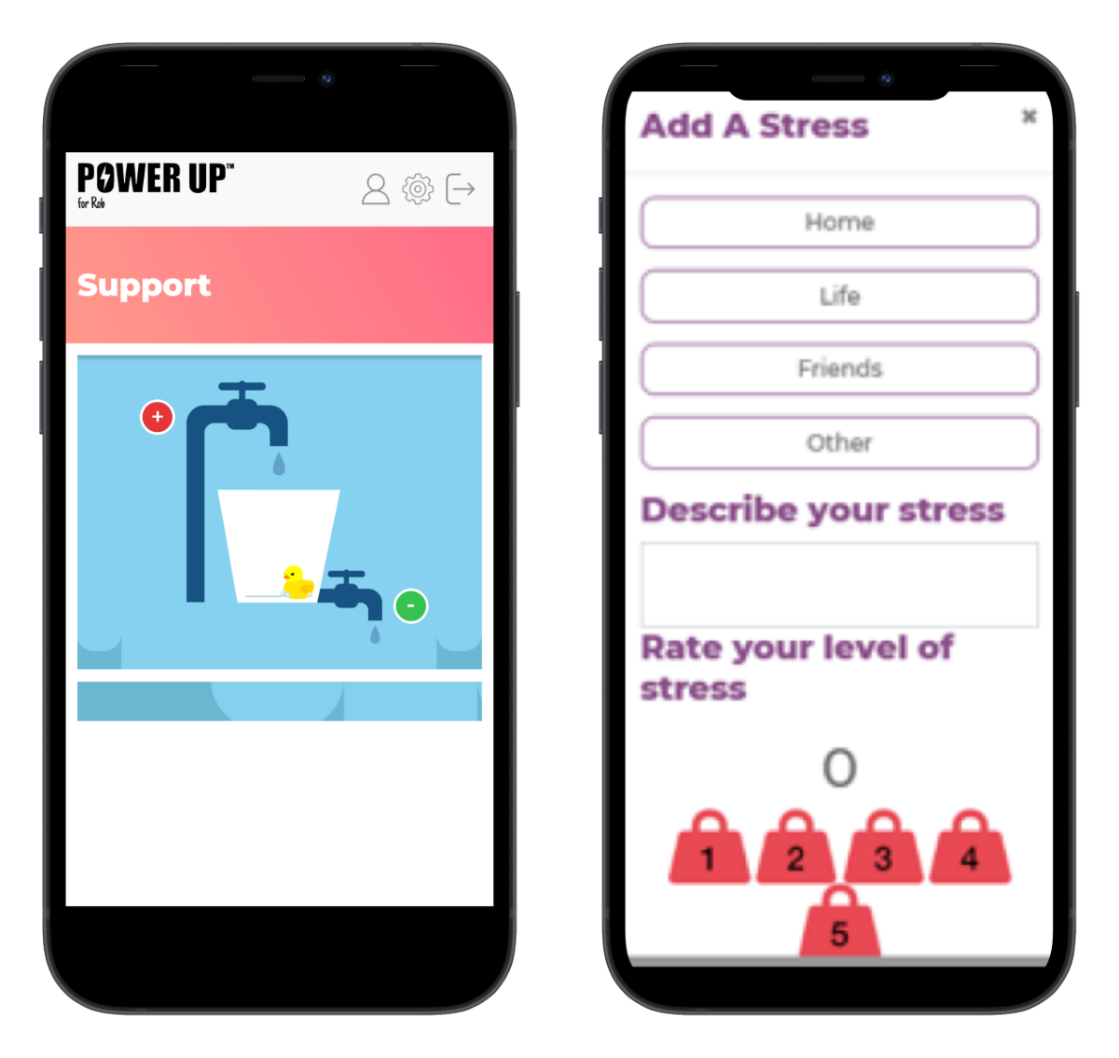
Examples of the support tab.

This section hosts a tool to allow parents to identify and express their views on various stressors affecting the decision-making process. Users are encouraged to think about stressful things and explore ways to manage them. They are able to track feelings toward decisions and explore where additional emotional support is required (Figure 4). The stress bucket concept has been endorsed across health care and well-being settings with positive feedback across age groups [50].

#### Resources

**Figure 5:**
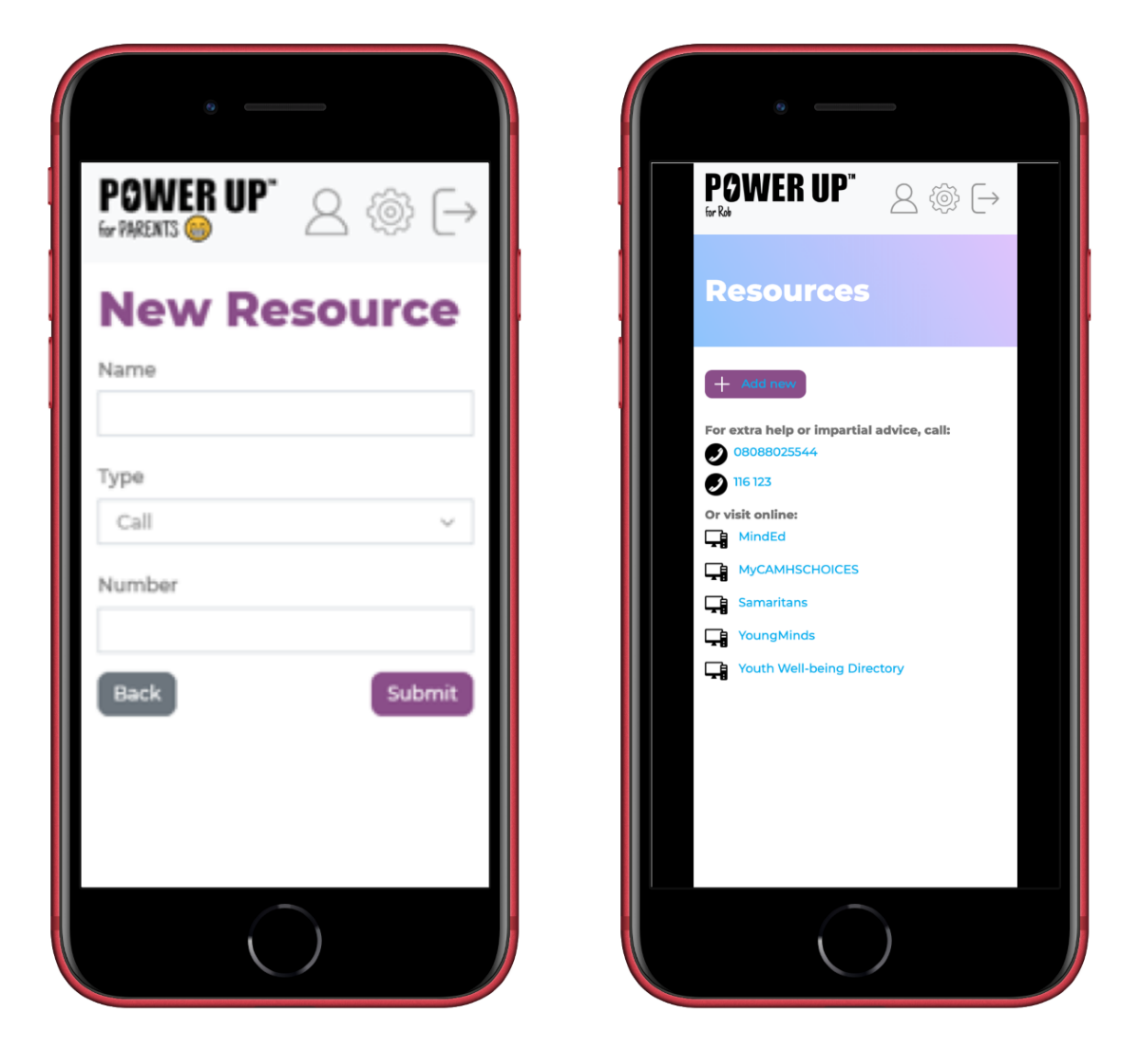
Examples of the resources tab.

This section includes useful contact details that signpost users to provide further support and guidance. Parents could upload their own resources to help with the decision-making process and include contacts they find most helpful (Figure 5). Parents involved in previous child and adolescent mental health research indicated the benefits of receiving information and expressed feeling more included when provided with adequate evidence [7]. However, parents reported feeling overwhelmed when too much information was given at once. This section allows parents to work with service providers to identify and obtain tailored resources.

## Discussion

### Principal Findings

This paper describes an evidence-based process for the development of a complex intervention—referred to as Power Up for Parents—based on the MRC framework [33] and guided by the workbook for developing and evaluating decision aids [34]. Stakeholder input from parents and carers, service providers, researchers, and young service users informed the design and content of the intervention. The intervention was developed in accordance with the International Patient Decision Aids Standards and guidelines [36,37] and grounded in the following four SDM models: the Youth SDM model [38], the Ottawa Decision Support Framework [39], the Integrative Model of SDM in medical encounters [29], and the affective-appraisal approach to SDM in CAMHS [13]. The objectives of the intervention were informed by empirical studies and literature reviews, which highlighted the need to provide additional support for parents of children with mental health problems who are involved in child mental health decisions [13]. The resulting prototype aimed to (1) encourage discussion, (2) allow parents to ask questions during sessions or seek further information within sessions, (3) provide a space for parents to identify their own feelings or moods and receive support, and (4) allow service providers to tailor the SDM process to accommodate the needs of the parent and child. To address the design aims, five key sections were embedded into the intervention. These features were the *Decisions*, *Goals*, *Journey*, *Support*, and *Resources* sections.

### Comparison With Existing Literature

The development process described in this paper is consistent with the development process briefly outlined in other parent-targeted SDM interventions [51-54]. Developers generally reported using end-user feedback, literature reviews, established guidelines, and empirical studies to inform the intervention. Overall, researchers have reported adopting one or a subset of these approaches to inform the intervention development process. However, only Hayes et al [54] reported using the MRC guidelines to inform the development of the i-THRIVE Grids. The current development process is in line with recommendations to promote well-being factors (eg, emotional regulation) in addition to targeted behavior change (eg, SDM) [23]. The iterative and collaborative approach adopted also supports other *user-based* frameworks embedded in human-computer interaction, such as the multiphase optimization strategy framework [55].

### Key Learnings

Common themes were identified across the steps of the development process of Power Up for Parents. These were interpreted to develop a list of eight recommendations to inform policy and practice guidelines. The recommendations were initially developed by the primary author and reviewed using an iterative process by 6 independent reviewers (ie, 2 practitioners, 1 child development policy officer, 1 parent with experience of having a child with mental health problems, and 2 child mental health researchers) before reaching a consensus to include. In line with the Salzburg statement on SDM [32], the following eight perceived key learnings were highlighted:

1. Ensure that primary carers and young service users are invited to be part of the care and treatment decision-making process while considering the following: the age and capacity of the child; how much the child wishes to have the parent involved or informed; and how much or what support the family needs to be involved.
2. Review clinicians’ time schedules so that they can provide sufficient time and encourage primary caregivers to ask questions and raise concerns during and within sessions.
3. Highlight the need for emotional support to be provided to primary caregivers, especially at the initial stages of accessing CAMHS or at crucial decision-making time points.
4. Propose a need for a key person in CAMHS who can provide answers to more general questions or be a liaison between clinicians and families, especially during periods when there is a change in service providers.
5. Consider the inclusion of the primary caregiver or key person (ie, an advocate for the family who is not the primary service provider) at multidisciplinary meetings when care and treatment options are being considered.
6. Review the role of parent support groups and explore the potential for further responsibilities.
7. Highlight the need for SDM support interventions as an adjunct to routine care.
8. Consider PPI activities at the core of design, development, testing, and implementation when SDM interventions are being developed. In doing so, it is also important that equal voices are given to service users and service providers, while ensuring interventions are accessible, acceptable, suitable and appropriate for the population, easy-to-use, useful, and do not incur additional time burden to service providers and service users.

### Implications for Implementation Science

Interventions addressing mental health concerns or SDM could replicate this development process if the intervention was found to be effective in later studies. With the high prevalence of child mental health problems and the alarming emotional state of parents, CAMHS could benefit from offering web-based support to parents in the absence of resources to facilitate face-to-face sessions with such large numbers of families. In addition, developing an intervention that encourages service users to collaborate with service providers can empower service users.

### Implications for Research

In keeping with the MRC framework, the intervention then entered the pilot and feasibility phase for testing the intervention, as discussed in the study protocol [56]. Preliminary results of the feasibility study [57] indicated that the intervention itself is generally acceptable by parents, carers, and health care professionals. The findings also indicate that there is scope for further development of Power Up for Parents. Results from the feasibility and pilot study have been integrated into refinements of the intervention and plans for further research.

### Strengths and Limitations

First, the main strength of this development process is the adoption of participatory design methods, where researchers, app developers, service providers, parents and carers, and young people were involved as partners at various stages to determine the content and design of Power Up for Parents. Second, adhering to the MRC framework and following the workbook for developing decision aids provided a solid foundation for evidence-based intervention. In addition, the theoretical underpinning and evidence base informing the content of Power Up for Parents provide a basis for potential success when the intervention is tested for effectiveness in future studies. Another strength is the dynamic nature of web applications to integrate into electronic health record systems or be embedded in National Health Services’ websites if found to be effective. Finally, the incorporation of all nine elements of SDM instead of the average 4.57 that is contained in similar interventions was viewed as a major strength.

However, the complexity of the intervention and the comprehensive approach taken to inform development resulted in a process that lasted almost 28 months. Although this may be viewed as a time-consuming process, developers aiming to develop similar interventions can use fewer empirical studies and incorporate rapid prototyping techniques [58]. In hindsight, another possible limitation could be the selection and combination of SDM models and theories. Other researchers in the field of SDM may criticize the chosen models and have a preference for alternatives. However, for the purpose of this research project, they seemed appropriate, and because they overlapped in some areas, they were readily combined. Similarly, the parents and young persons involved in the PPI sessions could represent a biased sample of persons who volunteered their time and expertise to inform research [59]. Therefore, they may not provide a broad representative view of families having a child with mental health problems. Moreover, the development of digital interventions can be costly. For this reason, it is recommended that cost-effectiveness be integrated into future study designs when evaluating interventions. Once proven effective, the cost can be justified as digital interventions have the ability to be scalable, affordable, and easily accessible to users [60-62]. Finally, the key learning and recommendations were based on a synthesis that went beyond the individual steps in the development process and a brief consultation exercise, and as such should be taken with caution.

### Conclusions

A multidimensional process was adopted, including an in-depth exploration of existing literature, empirical studies, theoretical underpinnings, and patient and public input to develop an evidence-based intervention to support parents involved in child and adolescent mental health decisions. The resulting intervention demonstrates and confirms that it is possible to use input from end users, integrated with theory and research evidence to create digital health interventions to be used in CAMHS. The intervention then entered the pilot phase aimed at obtaining end-user input for further development, views on acceptability, and an exploration of the feasibility of conducting a randomized controlled trial. The lessons learned from this process may inform the development of other interventions.

## Appendix

Multimedia Appendix 1 Logic model outlining the intervention process. CAMHS: child and adolescent mental health services; NHS: National Health Service; SDM: shared decision making.

Multimedia Appendix 2 User manual.

## Abbreviations

CAMHS: child and adolescent mental health services
MRC: Medical Research Council
PPI: patient and public involvement
SDM: shared decision making
