# Supplementary figures and images for: A Caregiver Digital Intervention to Support Shared Decision Making in Child and Adolescent Mental Health Services: Development Process and Stakeholder Involvement Analysis
Source: JMIR Form Res. 2021 Jun 15;5(6):e24896. doi: 10.2196/24896 (PMC8277368; doi:10.2196/24896)

# Logic Model

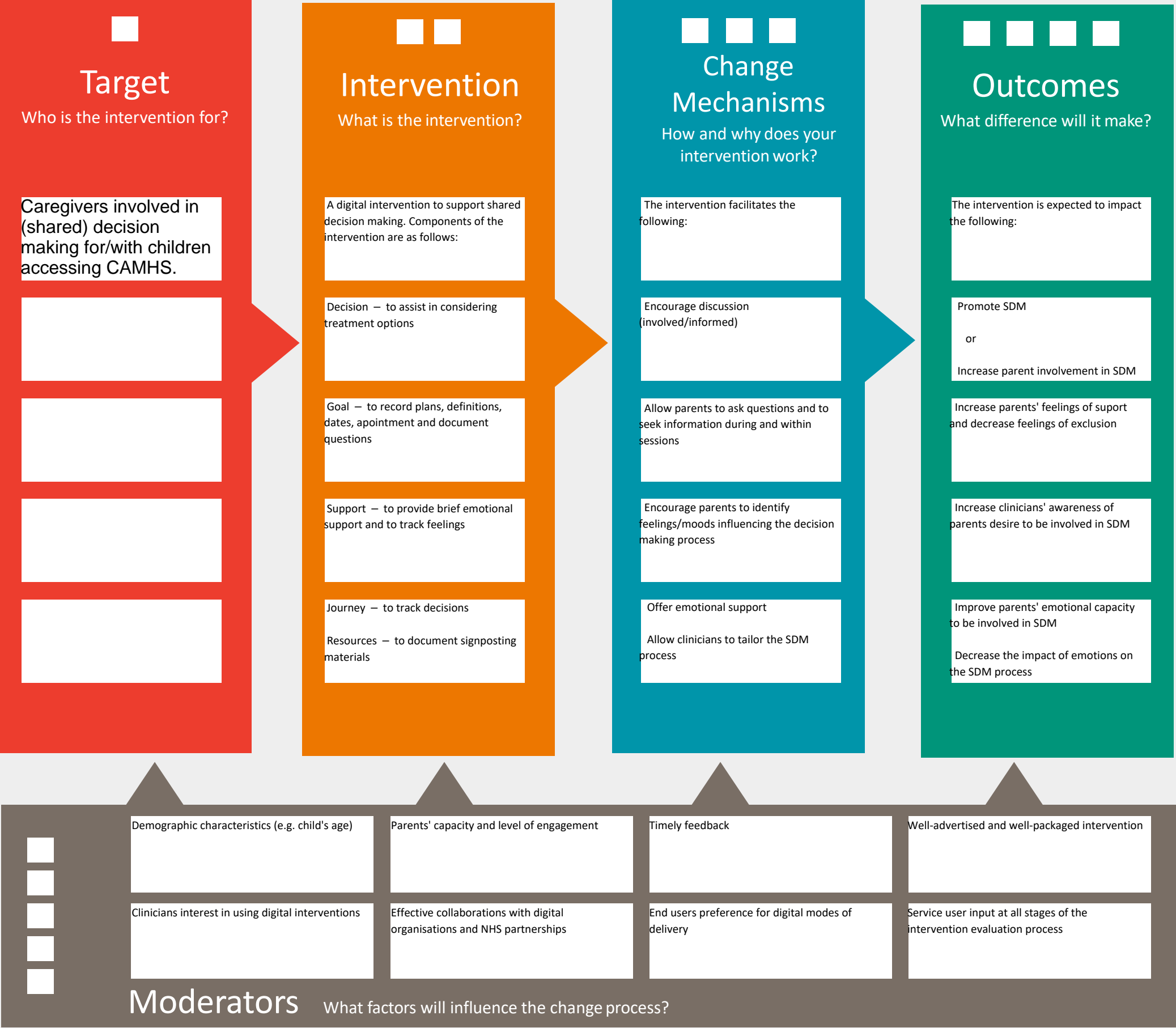

Supplement: Multimedia Appendix 1 [file formative_v5i6e24896_app1.pdf]
