# Supplementary material for: A Caregiver Digital Intervention to Support Shared Decision Making in Child and Adolescent Mental Health Services: Development Process and Stakeholder Involvement Analysis
Source: JMIR Form Res. 2021 Jun 15;5(6):e24896. doi: 10.2196/24896 (PMC8277368; doi:10.2196/24896)
Supplement: Multimedia Appendix 2 [file formative_v5i6e24896_app2.pdf]

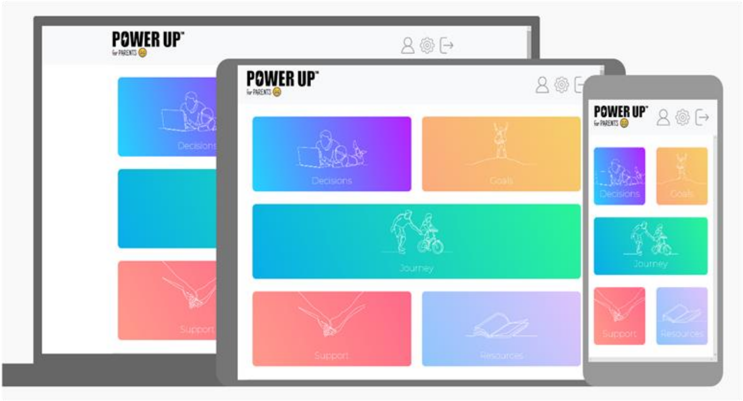

Power Up for Parents: A mobile application to support parents and primary carers making child mental health decisions

This work has been produced in part fulfilment of a PhD research project and sponsored by the **University College London**.

This research project is supported by **TEAM**, an Innovative Training network, funded by **European Union's Horizon 2020** research and innovation programme under the Marie Skłodowska-Curie grant agreement No. 722561.

This app was developed as a collaboration between researchers at the **Anna Freud National Centre for Children and Families**, University College London and **Create Health**.

For support or assistance needed while using this app please contact:

[redacted]

[redacted]

[redacted]

DRAFT

POWER UP FOR PARENTS

The Power Up for Parents app is designed to help facilitate the shared decision-making process. This app can support you and your family when making complex decisions about your child's or young person's (YP) mental health care and treatment.

As the primary carer, this app can help you decide what is most important to you and your family and encourage you to be more involved in care and treatment decisions.

Let's begin by getting you **signed in**:

1. First, enter an email address (This address will be used to send details if for some reason you forget your password and need to retrieve your account)

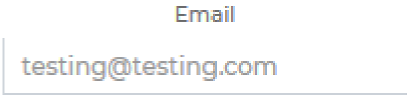

2. Then create a password (Ensure that it is memorable for you but difficult for others to decipher)

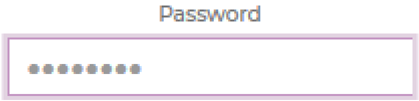

Now that you have signed in. Let's explore the main features of Power Up for Parents.

You can use the menu to customize your page.

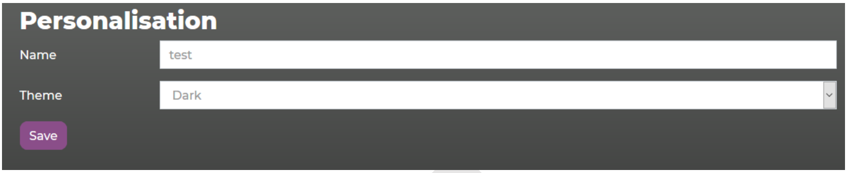

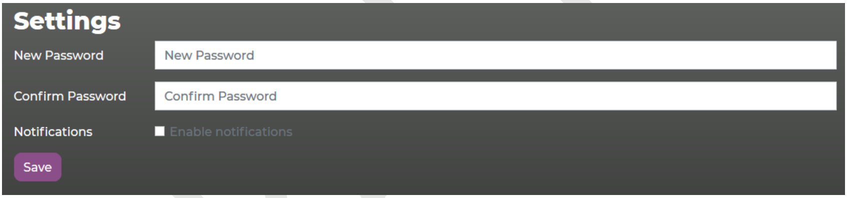

You can select any of the features for more guidance.

To return to this homepage at any time, you can click on the POWER UP™ logo.

To exit the app select the icon.

DRAFT

DECISIONS

Let us think through a decision that you may be faced with.

Consider that your child's health care professional suggested that you should start thinking of using medication for your child's challenging behaviour.

Click on the 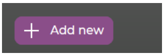

button.

Complete the details of the decision and select 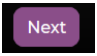

.

For example, Should my child take medication for challenging behaviours?

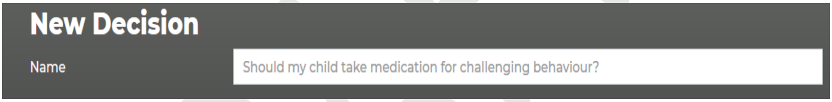

You may want to add some detail, so you remember why you are making this decision. You can enter this using the keypad or audio.

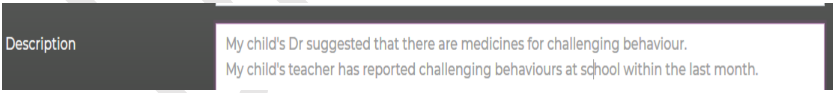

Indicate how important this decision is for you and your family (slide the bar).

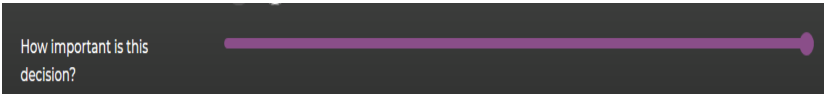

DRAFT

Think carefully about who else should be involved in this decision process? Using the 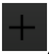, you can input the name and a photo (optional) of any others you choose to involve.

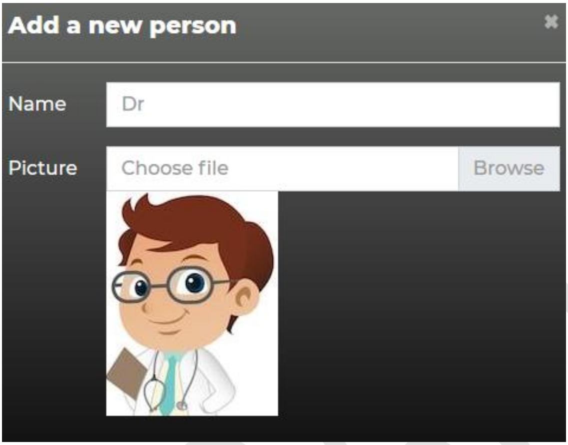

You may consider your child’s clinician for their expertise in mental health.

Depending on the age and capacity of your child you may want to include him or her as they are experts in their own lives.

It is recommended that you add a due date so you can receive reminders to complete or defer your decision as the date approaches.

Once you click 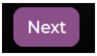

you are ready to compare the options.

DRAFT

In the chosen example, what are the options?

Click 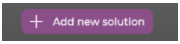

then 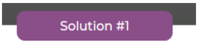

to enter the first option then 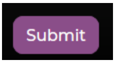

.

You may want to check to ensure these are all the options available to you and your family. Your child's health care professional may be able to confirm this.

Over the next few days you may want to go into each of these solutions and add the benefits and risks of each. You can do your own research. Ask health care professionals or seek out other parents for advice.

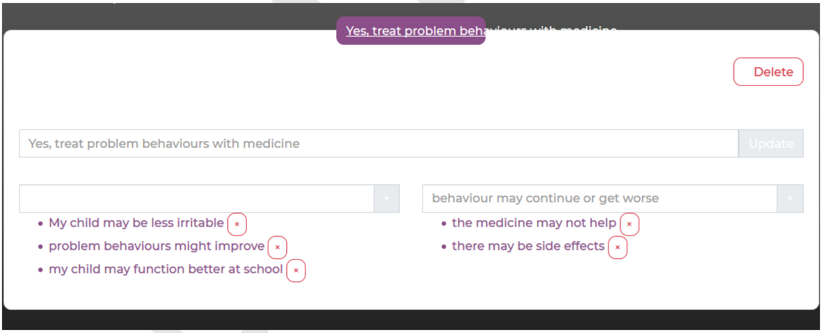

Then, you can select which of the options you prefer, and also take the opportunity to ask the other persons you involved for their preferences or recommendations.

Making a decision can be quite challenging. So it good to monitor your emotions throughout the process. You may want to give some detail about how you feel.

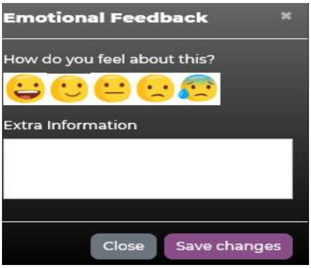

Each time you get new information you may need to revisit and enter new information or update old ones.

Once you are ready to may a decision select 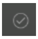 from the 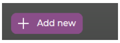

page. Your options are ranked in order, starting with the option with the highest number of pros to the top of the list.

However, this does not mean #1 may be the best option for you and your family at this time. For example, you may want to review which ones also had more preferences or the least number of cons.

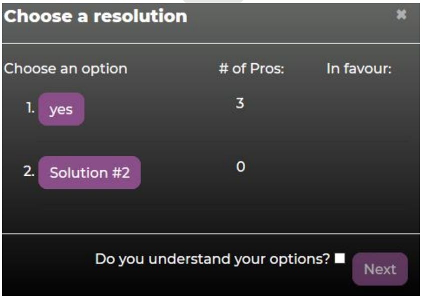

DRAFT

Now you can select which option you are most comfortable with. To ensure you are happy with your decision, the app will check if you understand the decision you are making and how you feel about it.

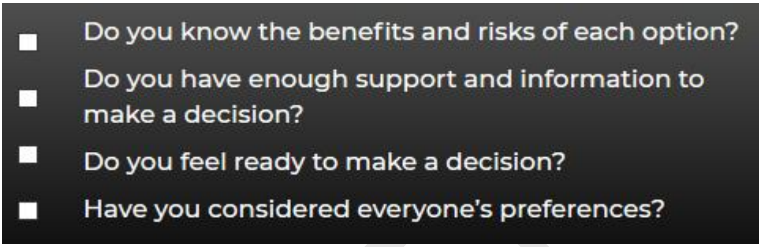

GOALS

You may want to link a goal to your decision or set a goal for yourself, your child or your family to accomplish.

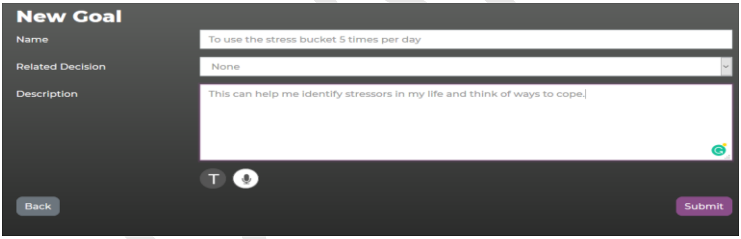

This goal can be linked to the decision on medications as this is one step you think may help you get closer to this goal. However, you can have general goals that are not necessarily linked to a decision.

You may want to use this feature to monitor any progress towards the overall goal. For example, you can set a goal to use the stress bucket feature 5x per day or once per week; whatever works for you.

DRAFT

SUPPORT

Sometimes difficult moments may come up. Imagine there's a bucket you carry with you which slowly fills up when you experience different types of stress. Now imagine you have a bucket and all these stresses fall into this bucket in the form of water.

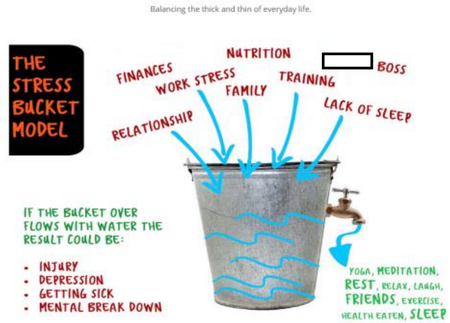

You may want to add your stressors using the 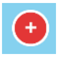; and think of what helps you reduce stress here using the 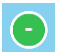 icon.

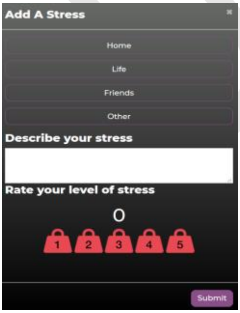

Remember to rate your stressors to help you monitor your stress levels.

DRAFT

RESOURCES

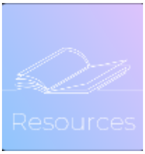

There are some useful contact details in this section to access further support and guidance if needed.

Feel free to add any resources of your own that you may find helpful. You can also upload images and documents that help you understand your child's mental health problems better.

JOURNEY

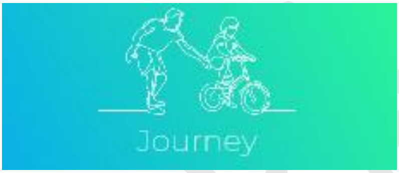

This is where you come to have a look back at your journey throughout the decision-making process. You may want to share this with a trusted friend or someone who supports you during difficult times.

You can also use the 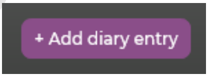

to add more details of your feelings and any challenges you face surrounding your child's condition. Remember you are not alone on this journey and there may be some resources your child's health care professional can suggest.

DRAFT

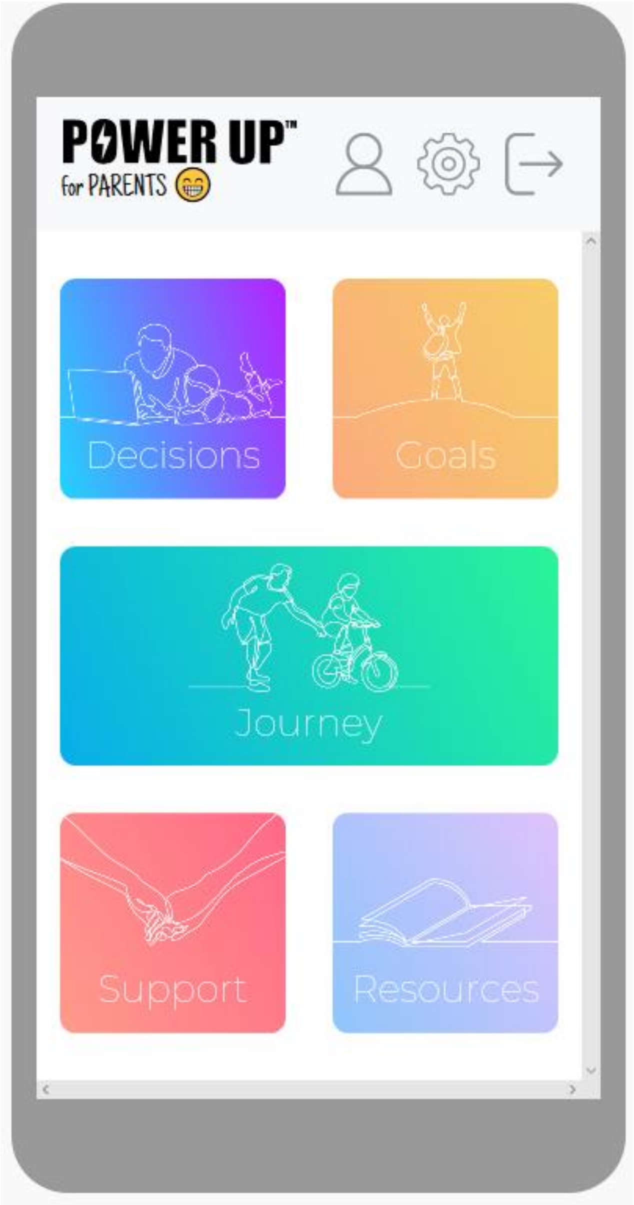
POWER UP™
for PARENTS
Decisions
Goals
Journey
Support
Resources
